# P02.59. Complexity of disease and population heterogeneity require a rethinking of clinical trial design

**DOI:** 10.1186/1472-6882-12-S1-P115

**Published:** 2012-06-12

**Authors:** H Amri, A Haramati, M Abu-Asab

**Affiliations:** 1 Georgetown University Medical Center, Washington, D.C., USA; 2 National Institutes of Health, Bethesda, USA

## Purpose

Although randomized clinical trials are considered the gold standard in biomedicine, they have several limitations such as experimenting on a sample group under ideal set forth criteria, creating an environment that does not apply to daily clinical practice where comorbid conditions are encountered everyday, and ignoring the differential response to treatment and treatment-related risk detected in the study population. This is due in part to the inherent complexity of the disease process and the heterogeneity within and between populations -- two fundamental principles of biology that currently do not receive sufficient attention in study design. The advent of high throughput technologies of genomics, proteomics, and deep sequencing has demonstrated the heterogeneous nature of biological data. In this paper, we propose to demonstrate how high throughput data combined with evolutionary-based analyses could be used to stratify patients prior to starting the clinical study into groups that share similar traits and affinities. With this information in hand, participants can then be randomized to control or treatment groups within each phyletic clade (or population).

## Methods

The method combines omics data with parsimony phylogenetics which we developed and named Phylomics®.

## Results

This novel approach offers a four-stage clinical trial design: 1) recruitment from a heterogeneous population using a wide spectrum inclusion criteria to reflect “real world” settings; 2) randomization within homogenous phyletic groups; 3) classification of phyletic groups into responders and non-responders to drug/intervention; and 4) translation of the clinical trial findings to the clinic. By knowing the phyletic clade of the patient, the physician would prescribe the drugs that are most suitable for the patient, based on his/her membership to the phyletic clade.

## Conclusion

This methodology applies personalized medicine within a systems biology paradigm that utilizes data heterogeneity, monitors treatment response and treatment-related risks, and facilitates a better translation of trials’ results to the clinic.

